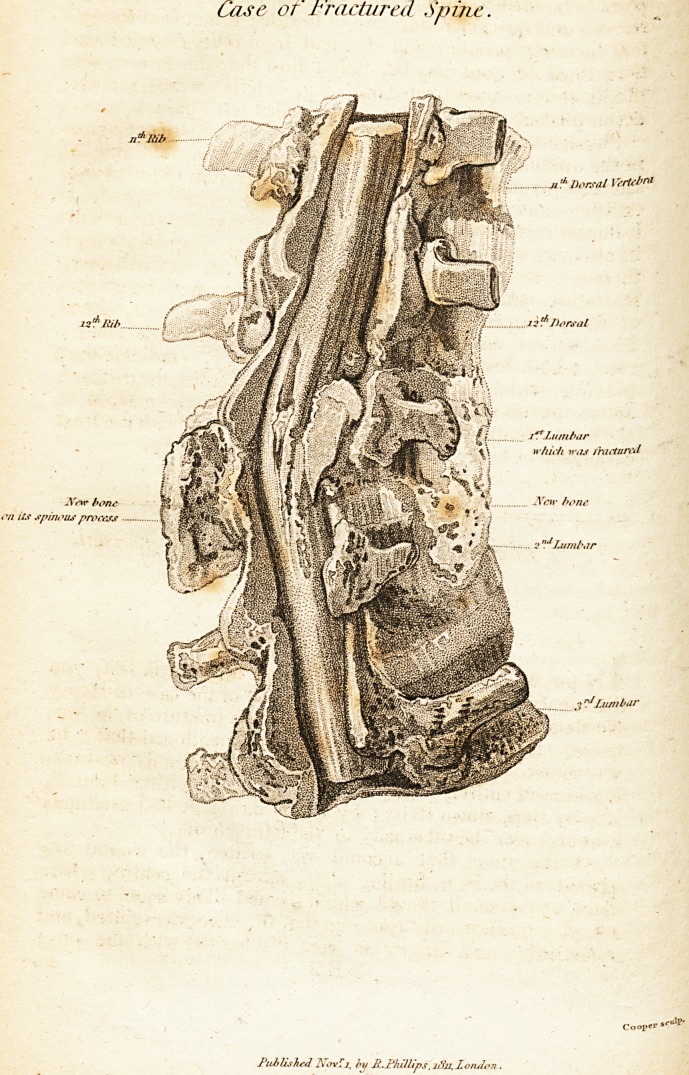# Mr. Harrold's Case of Fractured Spine

**Published:** 1811-11

**Authors:** E. Harrold

**Affiliations:** Cheshunt, Herts


### Case of Fractured Spine.

### *To the Editors of the Medical and Physical Journal.*

(With an Engraving.)

Gentlemen,

In the Medical and Physical Journal for March last, you did me the favour to publish an account of the case of Henry Newland, who was under my care with a fracture of the vertebræ. Near the end of this history I mentioned that "he was so active as to have made a practice, not only of dressing himself entirely, but, for some time, of letting himself, step by step, down stairs; by which means he had produced a wound over the tuberosity of the left ischium."

At the time that account was written, the wound appeared to be in a healing state, except the centre, where there was a small slough which seemed likely soon to come away. It afterwards appeared that the *bone* was injured, and sometimes small fragments were discharged with the pus;

 no no other application was used than a common poultice. About the middle of May last, hearing that he was very ill, I called to see him, and found, upon inquiry, that the wound had been skinned over; that, in consequence, a considerable quantity of pus had collected, which, by the cicatrix giving way, had been discharged to the amount of a pint and a half. His nights were bad; there was a good deal of nausea and anorexia; his pulse weak, and the discharge considerable. I ordered some tonics for him with an opiate at night, and a nourishing diet with wine. The external opening of the wound not being sufficient to allow a free exit to the matter, I enlarged it considerably: the divided part had the usual appearance of a fresh wound of a sound part, but he had not the slightest feeling of pain in the operation. From this time the discharge continued to be copious, with the usual smell of diseased bone; the powers of his stomach were never restored; constant pressure upon the right hip had destroyed the integuments of that also, and he died on the 29th of May, *just ten days less than a year after the fracture of the spine.*

I was anxious that Mr. Thomas Blizard should see the state of my patient after death, but his professional engagements at that time were too numerous to admit of his absence from town, and I proceeded to examine the body on the 31st.: I had not, however, time to extend my inquiries very far, but, I hope, sufficiently so to make the history instructive. On opening the abdomen the liver appeared paler than usual, but not diseased; the intestines had the usual appearance; the bladder contained about half a pint of urine, its coats a little thicker than natural: the kidneys and ureters healthy. On opening the thorax the lungs were uncommonly pale, or, rather white, as if they had been washed in milk, and on the right side adhered pretty generally to the pleura; the pericardium adhered to the whole surface of the heart.

In order to examine the spine more effectually, I removed six of the vertebræ, comprehending two above and two below those concerned in the fracture; the bodies of *these* had been evidently crushed, and had not quite recovered their original thickness or distinctness of articulation, but they were firmly and soundly united, bending a little forward, where the intervening cartilage had been obliterated, which accounts for the greater projection of their spinous processes, (as mentioned in the former part of the history) and inclining a little to the right side.

On viewing this detached part of the spine, the curvature and inclination, I should have supposed, would have produced in the living subject some degree of apparent deformity, which, though often looked for, was never observed. There was was some exuberance of boney matter where the bones were united, on the anterior side of the bodies of the vertebræ, inclining a little to the right side, in some extent caseing the original bone. The medulla spinalis appeared healthy and filled the canal at the upper part, above the injury, but at the lower orifice it seemed to me to be in some degree wasted, not filling the canal so compleatly there as above.

I sent the bones, in their undivided state, to Mr. T. Blizard, and through his kindness and that of my friend, Sir William Blizard, they are now deposited in the Museum of the Royal College of Surgeons in Lincoln's-inn-fields. The following is part of a letter which I received soon afterwards from Mr. T. Blizard.

"My dear Sir,—I have just returned from examining your "preparation. Mr. Clift (the conservator of the Museum) "has removed the spinous processes of the vertebræ, which "admirably displays the extent of the injury.

"The fracture was completely and firmly united; the pos-"terior part of the body of the fractured vertebræ was driven "into the medulla spinalis, its sheath being burst open, and "the medulla, at this part, was nearly destroyed. Above "and below the injury the appearance of the medulla was "natural. Under these circumstances it is hardly probable "that the parts supplied with nerves from the medulla, below "the injury, would ever have recovered the functions de-"pendant on nervous influence.

"When fracture of the spine takes place without any "actual pressure on, or laceration of, the medulla spinalis, "there is no reason why complete recovery should not take "place; and this even although paralysis should at first be "produced, which, as the consequence of concussion only, "may go off."

Since I received this letter, Mr. Clift has been so good as to show me the preparation in the Museum. The fracture appears to have extended in an oblique direction through *three* of the vertebræ, the lowest of the dorsal and the first two of the lumbar, and it seems to be a part of the body of the first lumbar vertebræ that was driven into and *remained* in the medulla spinalis; the canal is in a great degree narrowed at this part; but as Mr. Clift has been so obliging as to promise me two views of the preparation, I need not be more particular in my description of it.

Several questions naturally arise from the History of this case, and some useful deductions may be drawn from it. It is to be remarked that the *immediate* cause of my patient's death was not the first accident, but the second; not the fracture of the vertebræ, but the diseased state of the os ischium.

chium. How much longer he might have lived, had care been taken to prevent this last accident, it may, perhaps, be difficult to conjecture, for before he was prevented by it from being placed daily in the open air, he not only had the look and feeling of health but was gaining flesh.

I feel it to be my duty to correct a mistake in my first report of his case, where I remarked that he then began to be conscious of the passage of stools, except when they were liquid; farther inquiry did not confirm this opinion.

My patient, though he had never after the accident any sensation in the penis, felt pain when he compressed the testes, for which it is not difficult to account; but how are we to account for the pain, of which he often complained, in his right thigh?

Life having been preserved to so unusual length of time in this instance, which must be admitted to have been a fracture of the worst description, both in kind and degree, it becomes a matter of considerable importance to obtain information of the appearance on dissection of similar cases, to ascertain in what proportion of them the spinal marrow was actually wounded; to assist in forming our prognosis in future; and it will also be material to learn in what *manner* and to what *distance* the patient was conveyed after the accident, before he came under the Surgeon's care, as well as the mode of infliction of the injury in the first instance.

*My* patient was conveyed two miles in a heavy rough-shod cart, and it is fair to conclude that a ragged piece of bone, which might not wound at first, by rude and improper movement of the patient, might absolutely divide the medulla spinalis.

Should it turn out, upon making the inquiry which I have suggested, that, in many instances of fracture of the spine, the medulla is not absolutely *wounded*; there would then, I think, be a good prospect, under proper and careful management, not only of prolonging the life of the patient, but, in time, of restoring the use of the paralytic limbs. The prognosis must be formed not only from the particular symptoms of the patient, but from the result of the before mentioned *general* inquiry.

I shall now venture to propose a few hints for the guidance of practitioners in future, and in so doing, I shall be happy to stand corrected, should I be found in error, or to receive any additional information from your numerous and intelligent Correspondents.

The first rule is that the patient should not be moved the *shortest* distance, if it can be prevented, before he be properly secure secure upon the fracture-bed, and in the act of moving him into the fracture-bed, the greatest care must be taken to disturb the spine as little as possible.

2dly. The patient should be laid, of course, upon his back; and after his lower limbs are properly disposed in the fracture boxes, (to which none but the *outside* thigh splinter will be wanted) a little extension should be made by drawing him gently upwards towards the head of the bed.

3dly. The shoulders should be very little, *if at all*, elevated, as this may tend to produce some curvature of the spine, and to increase, or occasion pressure upon the medulla.

4thly. The catheter should have a tube of similar diameter made to fit into it easily after its introduction, at a right angle, which, by passing downwards between the thighs, may act as a syphon, and thus not only completely empty the bladder, but render pressure upon the abdomen unnecessary, and to prevent the mattress being wetted, which would otherwise be avoided only by taking it away.

5thly. It may be found useful to support the back on each side of the fracture by carefully pressing under a small soft cushion on each side. In Newland's case a worsted stocking twice doubled answered the purpose.

6thly. To save the small mattress which lies upon the trap-door, it will be adviseable to cover it with oiled silk.

Since the former part of this communication was written, I have been favoured with a letter from Mr. Clift, and a very beautiful drawing of the preparation of the spine. As some description accompanies the drawing, I need not say much about it. The fourth lumbar vertebra has been omitted, which, as Mr. Clift remarks, had nothing to do with the disease, and the omission allows the other parts to be represented of their natural size.

The drawing gives a view of the medullary canal, the spinous processes having been removed by the saw, and a part of the body of the first lumbar vertebra is very distinctly seen penetrating through the medulla spinalis.

Having, within these few days, sent an exfoliated *os pubis* to the Museum of the Royal College of Surgeons, and as the following letter to Mr. Clift contains a short history of the case, you will oblige me by inserting it in your valuable Journal.

"Dear Sir,

"When I had the pleasure of seeing you in the College Museum, I mentioned a case of fracture of the right os pubis, the heads of it are as follow.

"Anne Hill, five years old, on the 5th of August, 1808, had this bone fractured by the fore-wheel of a loaded narrow-wheeled waggon as she lay, thrown down upon the ground, ground, upon her back; the wheel passed between her legs, over the right os pubis, and, apparently, in that direction over the hip, when she was disengaged.

"On examination, the right os pubis was depressed entirely out of the reach of the finger. She recovered without any marked symptoms, her knees at first being kept together and bent, to prevent any unnecessary motion. A small wound was soon produced in the groin by inflammation, sufficient for the discharge of pus. On the 27th of October following, one part of the fractured bone was discharged, and in the course of a few weeks more the other part.

"The bone appears to have been fractured about the centre, and detached at the two extremities; and the two pieces discharged, or exfoliated, seem to comprehend almost the whole of the bone from the symphisis pubis to the acetabulum. The accident, after the bone was discharged, was followed by no lameness or inactivity. The child is now eight years old, rather delicate, which she has always been, but active. There is a considerable degree of fullness of the part; from the want of the boney support, it yields readily to pressure, and there is no evidence of the reproduction of bone.

"Should you think this exfoliation, with the summary history of the case above detailed, worthy a place in the College Museum, do me the favour to place it in that noble collection; and, with your permission, that the record of the case may be more generally known, I will send a copy of this letter, for publication, to the Editors of the Medical and Physical Journal.

"E. Harrold."

*Cheshunt, 3d Sept.* 1811.

The following Postscript was at the bottom of the letter in which Mr. Clift did me the favour to inclose the drawing, and acknowledges the receipt of the above letter and the preparation.

"I received your favour of the 3d inst. safe, and shall take "care to present it at the next Board of Curators. I think it "an admirable illustration of what nature will accomplish "for the recovery of a part, and what a degree of injury "may be sustained without proving fatal, particularly in "young subjects."

It will, I think, be interesting to observe the progress and effect of pregnancy in this case, should my patient live to be in that situation.

I have the honour to be,
Gentlemen,
Your obliged humble Servant,
E. HARROLD.

## Figures and Tables

**Figure f1:**